# A pooled analysis of six large-pen feedlot studies: effects of a noncoated initial and terminal implant compared with a single initial and delayed-release implant on arrival in feedlot heifers

**DOI:** 10.1093/tas/txaa109

**Published:** 2020-07-02

**Authors:** Zachary K Smith, David G Renter, Ben P Holland, Alyssa B Word, Grant I Crawford, Wade T Nichols, Brandon L Nuttelman, Marshall N Streeter, Lee-Anne J Walter, John P Hutcheson, Bill Dicke, Robert T Brandt, Josh I Szasz, Tony C Bryant, Lois F G Pringle, Zac E Carlson, Galen E Erickson, Bradley J Johnson

**Affiliations:** 1 Department of Animal Science, South Dakota State University, Brookings, SD; 2 Center for Outcomes Research and Epidemiology, Kansas State University, Manhattan, KS; 3 Cactus Research Ltd., Amarillo, TX; 4 Merck Animal Health, De Soto, KS; 5 Dicke Consulting, LLC, Lincoln, NE; 6 Brandt Nutrition Systems, LLC, Manhattan, KS; 7 Five Rivers Cattle Feeding, LLC, Johnstown, CO; 8 Department of Animal Science, University of Nebraska, Lincoln, NE; 9 Department of Animal and Food Science, Texas Tech University, Lubbock, TX

**Keywords:** beef, estradiol, feedlot, growth, heifers, trenbolone acetate

## Abstract

Randomized complete block design experiments (*n* = 6 experiments) evaluating steroidal implants (all from Merck Animal Health, Madison, NJ) were conducted in large-pen feedlot research facilities between 2015 and 2018 comparing an 80 mg trenbolone acetate (TBA) and 8 mg estradiol-17β (E_2_) initial implant (Revalor-IH) and reimplanted with 200 mg TBA and 20 mg E_2_ (Revalor-200; REPEATED) to a single 80 mg TBA and 8 mg E_2_ uncoated; 120 mg TBA and 12 mg E_2_ coated implant (Revalor-XH) at arrival (SINGLE) on growth and carcass responses in finishing heifers. Experiments occurred in Nebraska, Oklahoma, Washington, and Texas. Similar arrival processing was used across experiments where 17,675 heifers [initial body weight = 333 kg SEM (4.1)] were enrolled into 180 pens (90 pens per treatment with 65–240 heifers per pen) and fed for 145–222 d. Only REPEATED heifers were removed from their pen at reimplant. Diets contained monensin and tylosin, consisted of ingredients common to each region, and contained greater than 90% concentrate. Ractopamine hydrochloride was fed for a minimum of 28 d prior to harvest. Linear mixed models were used for all analyses; model-adjusted means for each implant group and the corresponding SEM were generated. Distributions of U.S. Department of Agriculture (USDA) quality grade (QG) and yield grade (YG) were analyzed as ordinal outcomes. No differences (*P* ≥ 0.11) were detected for any performance parameters except dry matter intake (DMI), where SINGLE had greater (*P* = 0.02) DMI (9.48 vs. 9.38 ± 0.127 kg) compared with REPEATED. Heifers implanted with REPEATED had greater (*P* ≤ 0.02) hot carcass weight (HCW; 384 vs. 382 ± 2.8 kg), dressing percentage (64.54 vs. 64.22 ± 0.120%), and ribeye area (91.87 vs. 89.55 ± 0.839 cm^2^) but less (*P* ≤ 0.01) rib fat (1.78 vs. 1.83 ± 0.025 cm) and calculated YG (2.82 vs. 2.97 ± 0.040) and similar (*P* = 0.74) marbling scores (503 vs. 505 ± 5.2) compared with SINGLE heifers. Distributions of USDA YG and QG were impacted (*P* ≤ 0.03) by treatment such that REPEATED had fewer USDA Prime and YG 4 and 5 carcasses. Heifer growth performance did not differ between implant regimens, but HCW and muscling did, perhaps indicating that REPEATED may be suited for grid-based marketing, and SINGLE might be suited for heifers sold on a live basis depending upon market conditions and value-based grid premiums and discounts. However, these decisions are operational dependent and also may be influenced by factors including animal and employee safety, stress on animals, processing facilities, time of year, labor availability, and marketing strategies.

## INTRODUCTION

Cattle implanted with a combination of trenbolone acetate (TBA) and estradiol-17β (E_2_) steroidal implants have been shown to consistently exhibit improved average daily gain (ADG), increased dry matter intake (DMI), and decreased dry matter (DM) feed conversion, marbling, and yield grade (YG) compared to nonimplanted cattle or cattle implanted with lower potency implants, fed for slaughter to equal days on feed (DOF; Johnson et al., 1996; Parr et al., 2011a; Smith et al., 2019a). Estrogenic implants change frame size and delay fattening in cattle (Preston, 1975). This shift in frame size and, ultimately, final shrunk body weight (BW) requires implanted cattle, and cattle given greater doses of anabolic compounds, be fed to greater final BW in order to reach similar empty body fat percentage as compared to cattle not administered a steroidal growth implant (Preston et al., 1990). No equivalent technology is available to producers that match the improvements in animal performance and hot carcass weight (HCW) delivered via implants at equal rib-fat accumulation (Reinhardt, 2007; Johnson et al., 2013; Johnson and Beckett, 2014). The primary objective of these experiments was to compare animal growth performance and carcass traits between a noncoated initial and reimplant program and a single-coated implant at feedlot arrival in beef heifers using a pooled analysis approach. A pooled analysis approach has many advantages over a single-trial analysis, such as a greater sample size to compare outcomes and also that a pooled analysis provides estimated means and the associated SEMs with more potential generalizability as they represent data from multiple populations of cattle, study sites, genetic types, and dietary ingredients.

## MATERIALS AND METHODS

### Animal Care and Use

The following experiment was a collaborative effort between Merck Animal Health, Cactus Research, Five Rivers Cattle Feeding, LLC, Dicke Consulting, LLC, Midwest PMS, LLC, South Dakota State University, Texas Tech University, University of Nebraska—Lincoln, and Kansas State University. Institutional Animal Care and Use Committee (IACUC) approval was not obtained at South Dakota State University, Texas Tech University, or Kansas State University and IACUC approval was obtained by the researchers at the University of Nebraska—Lincoln for the Nebraska study; all research herein was conducted at commercial research facilities and followed the guidelines stated in the Guide for the Care and Use of Agricultural Animals in Agricultural Research and Teaching (FASS, 1999).

### Trial Location, Animal Processing, and Feeding Management Details

Large-pen feedlot studies (*n* = 6) were conducted across the United States between 2015 and 2018 comparing a Revalor-IH and reimplant with Revalor-200 protocol to Revalor-XH in heifers fed in confinement for greater than 150 d. Trials were conducted (Table 1) in Nebraska (*n* = 1), Oklahoma (*n* = 1), Washington (*n* = 1), and Texas (*n* = 3), and all heifers used in these experiments were of breed type respective of their feeding region. All heifers were vaccinated and treated for internal and external parasites according to processing protocols at each specific feed yard. A total of 17,675 heifers were enrolled into 180 pens (*n* = 90 pens per implant program), pen sizes ranged from 65 to 240 heifers per pen, and DOF ranged from 145 to 222. Heifers in the Revalor-XH treatment group remained in their home pens from start to finish, while heifers that were reimplanted were removed from their pen on the day of reimplanting. Diets fed in these studies consisted of common ingredients to the specific geographic area of the commercial feedlot and contained greater than 90% concentrate. Ractopamine hydrochloride was fed to all heifers for a minimum of 28 d prior to harvest.

**Table 1.** Description of large-pen feedlot studies included in the pooled analysis^*a*,*b*^<?landscape?>

Location	Cattle type	Treatment structure	Treatments^*c*^	Reimplant date^*d*^	Replicate pens (blocks)/treatment	Heifers/pen	Initial BW, kg	Average DOF
Nebraska	Continental × British	Implant only	IH/200 and XH	101	6 (6)	73	322	183
Oklahoma	Continental × British	Implant only	IH/200 and XH	90	12 (12)	130	324	184
Washington	Continental × British	Implant only	IH/200 and XH	70	8 (8)	221–240	401	153
Texas	Continental × British	Implant only	IH/200 and XH	71	10 (10)	125	317	164
Texas^*e*^	Continental × British	Implant × DOF	IH/200 and XH; 172, 193, or 214 DOF	90	27 (9)	70	309	193
Texas^*e*^	Continental × British and British × *Bos Indicus*	Implant × DOF	IH/200 and XH; 150, 171, or 192 DOF	90	27 (9)	65–70	337	171

^*a*^Enrolled heifers = 17,675, pens = 180, blocks = 54, studies = 6; DOF range = 145–222, mean = 180 DOF.

^*b*^The Oklahoma study included: Revalor-200/Revalor-200 and Synovex ONE-Feedlot groups that were not included in the present analysis.

^*c*^Treatments used in the analysis were: Revalor-IH (80 mg TBA and 8 mg E_2_, uncoated) on arrival followed by Revalor-200 (200 mg TBA and 20 mg E_2_, uncoated) at approximately 70–101 DOF; or Revalor-XH (80 mg TBA and 8 mg E_2_, uncoated; 120 mg TBA and 12 mg E_2_, coated; 200 mg TBA and 20 mg E_2_, total) on arrival.

^*d*^Heifers in XH were not removed from their pen at the time of reimplanting.

^*e*^From: Smith et al. (2019a).

Implants used in these experiments (all from Merck Animal Health, Madison, NJ) included:

1) Revalor-IH (80 mg TBA and 8 mg E_2_) administered on arrival followed by Revalor-200 [200 mg TBA and 20 mg E_2_; (REPEATED)].
2) Revalor-XH (80 mg TBA and 8 mg E_2_ uncoated; 120 mg TBA and 12 mg E_2_ coated; 200 mg TBA and 20 mg E_2_ total) administered on arrival (SINGLE).

Heifers from SINGLE were not removed from their pens at any point during the course of the study; thus, no implant retention check occurred; heifers from REPEATED were subjected to an implant retention check during the reimplantation process; however, no implant retention check occurred following administration of the second implant.

### Study Design and Details: Nebraska

Starting in May of 2018, intact crossbred heifers (from four unique sources) were received from Nebraska (two replications), North Dakota, and Montana (two replications), as well as spayed feeder heifers from Canada (two replications) to a commercial research feedyard near Farnam, NE. At initial processing, heifers were vaccinated against viral respiratory pathogens (Bovi-Shield Gold 5; Zoetis, Parsippany, NJ) and the parasiticides oral fenbendazole (Safe-Guard; Merck Animal Health) and moxidectin pour-on (Cydectin; Bayer Animal Health, Shawnee, KS) were given for the control of internal and external parasites. Heifers were excluded from the study if they were considered unfit for the experiment (visually ill, lame, or of unlike breed type) or pregnant as identified via ultrasonography. A total of 870 heifers (initial allotment BW = 322 ± 8.9 kg) were blocked by arrival load (*n* = 6 blocks) and assigned randomly to one of two treatments as they passed through the processing shed, resulting in six pen replications per treatment (*n* = 73 heifers on average per pen and 435 heifers per treatment) and were used in a randomized complete block design. Heifers were housed in outdoor, soil-surfaced pens, with a concrete bunk apron, and pens were stocked so that each animal had approximately 7.1 m^2^ and 27.9 cm of bunk space.

Heifers were fed twice daily, and the transition to the finishing diet (93% concentrate) was done using a series of four step-up diets over a 27-d period. The finish ration was prepared in the on-site feed mill and contained 65.3% steam-flaked corn, 18.0% wet distillers grains plus solubles, 5.5% corn silage, 4.5% mixed hay, 1.7% tallow, and 5.0% supplement on a DM basis. Feed calls were made to provide feed to appetite and such that feed carryover in the bunk was minimized.

Monensin sodium (Rumensin, Elanco Animal Health, Greenfield, IN) was included in the finish ration to provide 340 mg/heifer/d throughout the experiment. Tylosin phosphate (Tylan, Elanco Animal Health) was included at 8 g/907 kg. To control the outbreak of heifers cycling, melengestrol acetate (MGA, Zoetis) was included in the diet to provide 0.45 mg/heifer/d. All heifers were fed 250 mg/heifer/d of ractopamine hydrochloride for 29 d with a 2-d withdrawal period prior to slaughter. Feed ingredients were collected weekly for DM determination on site, and these DMs were used for the calculation of DMI. The finishing diet provided protein and minerals to meet or exceed requirements (NRC, 1996).

Heifers were weighed as a group by pen using a platform scale at study initiation and the morning of shipment for the calculation of live growth performance. Body weights were measured prior to the morning feeding and a 4% pencil shrink was applied to the final BW measure, and carcass-adjusted performance was calculated from HCW/dressing percentage (DP) in decimal form.

Individual animal ID was tracked through the harvest floor, and HCW was captured as the carcass exited the harvest floor. Carcasses were graded after approximately 24-h chill. United States Department of Agriculture (USDA) quality grade (QG; assigned by USDA Grader) and YG (assigned by camera system), HCW, and grading camera measurements were obtained from the packing plant records. Dressing percentage for each pen was calculated as the mean HCW/final shrunk BW × 100.

### Study Design and Details: Oklahoma

Between January 25, 2018 and February 23, 2018, heifers were received at a commercial feed yard in Oklahoma. The yearling crossbred beef heifers were sourced from Arkansas, Colorado, Kansas, Mississippi, New Mexico, Oklahoma, and Texas. A total of 6,239 heifers were enrolled in the study. Heifers were arranged into blocks by arrival date (*n* = 12 blocks) and assigned to one of four implant treatments as they passed through the squeeze chute at initial processing, resulting in 12 pen replications per treatment (*n* = 130 heifers per pen and 1,560 heifers per treatment). The implant treatments included: 1) Revalor-IH (80 mg TBA and 8 mg E_2_, uncoated; Merck Animal Health) at initial processing and reimplanted with a Revalor-200 (200 mg TBA and 20 mg E_2_, uncoated; Merck Animal Health) after 90 d, 2) Revalor-200 at initial processing and reimplanted with a Revalor-200 after 90 d, 3) Revalor-XH (80 mg TBA and 8 mg E_2_, uncoated; 120 mg TBA and 12 mg E_2_, coated; 200 mg TBA and 20 mg E_2_, total; Merck Animal Health) at initial processing, or 4) Synovex ONE Feedlot (200 mg TBA and 28 mg estradiol benzoate, coated; Zoetis) at initial processing; only implant treatments 1 and 3 were used in the present analysis.

On the day of initial processing and randomization, heifers were excluded from the candidate population due to extremes in BW. Heifers were also excluded if they were considered unfit for the experiment (visually ill, lame, or of unlike breed type) or if they were determined to be bred at the time of processing. Initial processing also included vaccination against viral respiratory pathogens (Bovi-Shield Gold 5, Zoetis) and clostridial species (Vision 7, Merck Animal Health), as well as the application the of parasiticides injectable Ivermectin (Bimeda US, Oakbrook Terrace, IL) and Safe-Guard oral drench (Merck Animal Health) given for the control of internal and external parasites according to label instructions.

Heifers were housed in outdoor, soil-surfaced pens, and pens were stocked so that each animal had approximately 11.9 m^2^ and 23.1 cm of bunk space. Heifers were fed twice daily a milled ration consisting of steam-flaked corn (75.9%), wheat, and/or corn silage (9.2%), corn-milling byproducts (7.9%), and supplemental ingredients (7.0%). Heifers were fed a starter ration and were gradually adapted to a finish ration using a single intermediate ration and a series of step-up feeding schedules. Monensin sodium (Rumensin, Elanco Animal Health) was included in the finish ration at 34 g/907 kg (dry basis) and tylosin phosphate (Tylan, Elanco Animal Health) was included at 9 g/907 kg (dry basis). Additionally, MGA was included in the finish ration at a rate sufficient to provide melengestrol at 50 mg/907 kg (dry basis). Heifers were fed ractopamine hydrochloride at a target level of 27 g/907 kg (dry basis) for 34 d followed by a 4-d withdrawal prior to harvest. Feed ingredients were collected weekly for DM determination on site, and these DMs were used for the calculation of DMI. Feed calls were made to provide feed to appetite and such that feed carryover in the bunk was minimized.

Individual BW was recorded for each animal at the time of initial processing. Heifers were weighed as a group by pen using a platform scale the morning of shipment for the calculation of live growth performance. Body weights were measured prior to the morning feeding and a 4% pencil shrink was applied to initial weight and final BW. In addition, carcass-adjusted performance was calculated using carcass-adjusted final BW calculated from HCW/DP in decimal form.

All heifers were shipped by block to a commercial abattoir (JBS; Cactus, TX) as they became market ready from July to August 2018. Camera plant data was obtained, and DP for each pen was calculated as the mean HCW/mean final shrunk live weight (4% shrink) × 100.

### Study Design and Details: Washington

Between February 7, 2018 and March 3, 2018, a total of 3,686 heifers were transported 24 km from a backgrounding facility in Washington State. Heifers were of English and English × Continental breeding. At initial processing, heifers were vaccinated against viral respiratory pathogens (Vista 5, Merck Animal) and the parasiticides Safe-Guard oral drench and Synergized Delice pour-on (both Merck Animal Health) were given for the control of internal and external parasites. Heifers were excluded from the study if they were considered unfit for the experiment (visually ill, lame, or of unlike breed type) or bred, identified via ultrasonography; these heifers remained at the backgrounding yard and were not included in the potential pool of study candidates. A total of 3,686 heifers (initial allotment BW = 401 kg) were blocked by arrival date (*n* = 8 blocks) and assigned to one of two treatments as they passed through the processing shed, resulting in eight pen replications per treatment (*n* = 221–240 heifers per pen and 1,843 heifers per treatment) and were used in a randomized complete block design. Heifers were housed in outdoor, soil-surfaced pens, and pens were stocked so that each animal had approximately 18.6 m^2^ and 25.4 cm of bunk space.

Transition to the finishing diet was done using a two-ration approach. The finish ration was prepared in the on-site feed mill and contained (DM basis) steam-flaked corn (62.0%) and high-moisture corn (14.0%) in a 4:1 ratio, alfalfa hay (2.7%), triticale silage (6.1%) condensed corn distiller’s solubles (3.6%), canola meal (4.3%), tallow (3.2%), and a liquid supplement (4.2%). Monensin sodium (Rumensin, Elanco Animal Health) was included in the finish ration (36 g/907 kg) throughout the experiment. Tylosin phosphate (Tylan, Elanco Animal Health) was included at 8.3 g/907 kg. To control the outbreak of heifers cycling, MGA was included in the diet to provide 0.50 mg/heifer/d. All heifers were fed 19-g/907-kg ractopamine hydrochloride for 31–34 d prior to slaughter. All feed additives were included via a micromachine. Samples of each ration were collected daily from the feed bunk and dried at 100 °C. Daily DMs were averaged weekly and used for the calculation of DMI. The finishing diet provided protein and minerals to meet or exceed requirements (NRC, 1996).

Heifers were weighed as a group by pen using a platform scale at study initiation and the morning of shipment for the calculation of live growth performance. Body weights were measured prior to the morning feeding and a 4% pencil shrink was applied to final BW. In addition, the carcass-adjusted performance was calculated using carcass-adjusted final BW calculated from HCW/DP in decimal form.

Beef Carcass Research Center (West Texas A&M University, Canyon, TX) personnel recorded individual animal ear tag numbers in the sequence of harvest and affixed a harvest sequence number to each carcass. Plant carcass ID and HCW were recorded and verified by carcass sequence number during the harvest procedure. Carcasses were graded after approximately 36-h chill. United States Department of Agriculture QG (assigned by USDA Grader) and YG (assigned by camera system), HCW, and grading camera measurements were obtained from the packing plant records. Dressing percentage for each pen was calculated as the mean HCW/mean final shrunk live weight (4% shrink) × 100.

### Study Design and Details: Texas Study 1

Between January 18, 2018 and January 31, 2018, a total of 3,048 heifers were received at the commercial feed yard in Texas. Heifers were received from the Texas Panhandle, Oklahoma, Eastern New Mexico, and Kentucky and were mostly black-hided in color and of English and English × Continental breeding. At initial processing, heifers were vaccinated against viral respiratory pathogens (Vista 5, Merck Animal Health) and the parasiticides Dectomax Injectable (Zoetis) and Safe-Guard oral drench (Merck Animal Health) were given for the control of internal and external parasites. On the day of initial processing and randomization, heifers were excluded from the candidate population due to extremes in BW. Heifers were also excluded if they were considered unfit for the experiment (visually ill, lame, or of unlike breed type) or if they were determined to be bred at the time of processing. A total of 2,500 (initial allotment BW = 317 kg) heifers were blocked by arrival date (*n* = 10 blocks) and assigned to one of two treatments as they passed through the processing shed, resulting in 10 pen replications per treatment (*n* = 125 heifers per pen and 1,250 heifers per treatment) and were used in a randomized complete block design.

Heifers were housed in outdoor, soil-surfaced, pipe-constructed pens measuring 55 m deep and 30 m wide. Pens were stocked so that each animal had approximately 13.3 m^2^ of pen space and 24.4 cm of bunk space. Feed bunks were visually checked three times daily for the presence of residual feed. Feed calls were made to provide feed to appetite and such that feed carryover in the bunk was minimized.

The starter diet for all treatments was RAMP (Cargill Corn Milling, Bovina, TX). In addition, loose hay was top-dressed to the feed bunk for at least 3 d after arrival. Transition to the finishing diet was done using a two-ration approach where replacement of 10–15% of the daily feed call of RAMP was replaced with the finishing ration. Increases in the amount of finish ration were made every 2–4 d.

The finish ration was prepared in the on-site feed mill and contained (DM basis) steam-flaked corn (55.2%), Sweet Bran Plus (17.9%; Cargill Corn Milling), wet distiller’s grains plus solubles (17.2%), cotton burrs initially then ground cornstalks (7.2%), corn oil (1.5%), glycerin (1.0%), and microingredients (0.03%). Monensin sodium (Rumensin, Elanco Animal Health) was included in the RAMP (20 g/907 kg) and finish ration (42.1 g/907 kg) throughout the experiment. Tylosin phosphate (Tylan, Elanco Animal Health) was included at 9.6 g/907 kg. To control the outbreak of heifers cycling, MGA (Zoetis) was included in the diet to provide 0.40 mg/heifer/d. Feed calls were made to provide feed to appetite and such that feed carryover in the bunk was minimized. All heifers were fed 27.3-g/907-kg ractopamine hydrochloride for 29 d prior to slaughter. Samples of each ration were collected daily from the feed bunk and subsamples dried at 100°C. Daily DMs were averaged weekly and used for the calculation of DMI. The finishing diet provided protein and minerals to meet or exceed requirements (NRC, 1996).

Individual BW was recorded for each animal at the time of initial processing. Heifers were weighed as a group by pen using a platform scale on study day 0 and the morning of shipment for the calculation of live growth performance. Body weights were measured prior to the morning feeding and a 4% pencil shrink was applied to day 0 and final BW, and carcass-adjusted performance was calculated from HCW/DP in decimal form.

Trained personnel recorded individual animal ear tag numbers in the sequence of harvest and affixed a harvest sequence number to each carcass. Plant carcass ID and HCW were recorded and verified by carcass sequence number. Carcasses were graded after approximately 36-h chill. United States Department of Agriculture QG (assigned by USDA Grader) and YG, HCW, and actual and camera grading measurements were obtained from the packing plant records. Dressing percentage for each pen was calculated as the mean HCW/mean shrunk live weight × 100.

### Study Design and Details: Texas Study 2

Between July 22, 2015 and August 31, 2015, a total of 4,213 heifers were received at the commercial research feed yard in Texas. Heifers were of English and English × Continental breeding. At initial processing, all heifers were vaccinated against viral respiratory pathogens (Vista Once SQ, Merck Animal Health) and clostridia species (Vision 8, Merck Animal Health) and administered the parasiticides Cydectin Injectable (Bayer, Shawnee, KS) and Ultra Saber Pour-On (Merck Animal Health) for the control of internal and external parasites according to label directions. On the day of initial processing and randomization, heifers were excluded from the candidate population due to extremes in BW. Heifers were also excluded if they were considered unfit for the experiment (visually ill, lame, or of unlike breed type) or if they were determined to be bred at the time of processing. A total of 3,780 (initial allotment BW = 309 kg) heifers were blocked by arrival date (*n* = 9 blocks) and assigned to one of six treatments as they passed through the processing shed, resulting in nine pen replications per simple-effect treatment (*n* = 70 heifers per pen and 630 heifers per simple-effect treatment) and were used in a randomized complete block design. The study was originally designed as a 2 × 3 factorial treatment arrangement with three serial harvest dates and two implant treatments. Heifer growth performance and carcass traits from this experiment have been published previously (Smith et al., 2019a). No interactions were detected; therefore, only the main effect of implant across all harvest dates is represented in this paper.

The starter diet was RAMP (Cargill Corn Milling, Bovina, TX). In addition, loose hay was top-dressed to the feed bunk for at least 3 d after arrival. Transition to the finishing diet was done using a two-ration approach where replacement of 10–15% of the daily feed call of RAMP was replaced with the finishing ration. Increases in the amount of finish ration were made every 2–4 d.

The finish diet was prepared in the on-site feed mill and contained (DM basis): steam-flaked corn (48.5%), Sweet Bran Plus at 11.0% (Cargill Corn Milling), wet distiller’s grains plus solubles (34.2%), corn stalks (5.6%), yellow grease (0.72%), and microingredients (0.03%). Monensin sodium (Rumensin, Elanco Animal Health) was included in the RAMP (20.0 g/907 kg) and finish diet (42.0 g/907 kg) throughout the experiment. Tylosin phosphate (Tylan, Elanco Animal Health) was included at 9.6 g/907 kg. To control the outbreak of heifers, cycling MGA was included in the diet at 0.40 mg/heifer/d. All heifers were fed 27.3-g/907-kg ractopamine hydrochloride for approximately 28 d prior to slaughter (all feed additives on a DM basis). Feed calls were made to provide feed to appetite and such that feed carryover in the bunk was minimized. Samples of each ration were collected daily from the feed bunk and subsamples dried at 100 °C. Daily DM was averaged weekly and used for the calculation of DMI. The finishing diet provided protein and minerals to meet or exceed requirements (NRC, 1996).

Heifers were housed in outdoor, soil-surfaced, pipe-constructed pens measuring 53 m deep and 18 m wide in Exp. 1. Pens were stocked so that each animal had approximately 14 m^2^ of pen space and 25 cm of bunk space. Feed bunks were visually checked three times daily for the presence of residual feed. Feed calls were made to provide feed to appetite and such that feed carryover in the bunk was minimized.

Heifers were weighed as a group by pen using a platform scale on study day 0 and the morning of shipment for the calculation of live growth performance. Body weights were measured prior to the morning feeding and a 4% pencil shrink was applied to initial and final BW. Carcass-adjusted performance was calculated from HCW/DP in decimal form.

Trained personnel from the Beef Carcass Research Center recorded individual animal ear tag numbers in the sequence of harvest and affixed a harvest sequence number to each carcass. Plant carcass ID and HCW were recorded and verified by carcass sequence number. Carcasses were graded after chilling for approximately 36 h and USDA QG (assigned by USDA Grader) and YG (assigned by camera system) were obtained from the packing plant records. Dressing percentage for each pen was calculated as the mean HCW/mean shrunk live weight × 100.

### Study Design and Details: Texas Study 3

Between March 13, 2018 and May 3, 2018, a total of 4,233 heifers were received at the commercial feed yard in Texas. Heifers were of English and English × Continental and English × *Bos indicus* breeding. At initial processing, heifers were vaccinated against viral respiratory pathogens (Vista 5, Merck Animal Health) and the parasiticides Dectomax (Zoetis) and Safe-Guard oral drench (Merck Animal Health) were given for the control of internal and external parasites according to label directions. On the day of initial processing and randomization, heifers were excluded from the candidate population due to extremes in BW. Heifers were also excluded if they were considered unfit for the experiment (visually ill, lame, or of unlike breed type) or if they were determined to be bred at the time of initial processing. A total of 3,719 (initial allotment BW = 337 kg) heifers were blocked by arrival date (*n* = 9 blocks) and assigned to one of six treatments as they passed through the processing shed, resulting in nine pen replications per simple-effect treatment (*n* = 65–70 heifers per pen and 585–630 heifers per simple-effect treatment) and were used in a randomized complete block design. The study was originally designed as a 2 × 3 factorial treatment arrangement with three serial harvest dates and two implant treatments. Heifer growth performance and carcass traits from this experiment have been published previously (Smith et al., 2019a). No interactions were detected; therefore, only the main effect of implant across all harvest dates is represented in this paper.

Heifers were housed in outdoor, soil-surfaced, pipe-constructed pens measuring 53 m deep and 18 m wide. Pens were stocked so that each animal had approximately 14–15 m^2^ of pen space and 25–28 cm of bunk space. Feed bunks were visually checked three times each day for the presence of residual feed in the bunk. Feed calls were made to provide feed to appetite and such that feed carryover in the bunk was minimized from day to day.

The starter diet used in both experiments was RAMP (Cargill Corn Milling, Bovina, TX). In addition, loose hay was top-dressed to the feed bunk for at least 3 d after arrival. Transition to the finishing diet was done using a two-ration approach where replacement of 10–15% of the daily feed call of RAMP was replaced with the finishing ration. Increases in the amount of finish ration were made every 2–4 d.

The finishing diet was prepared in the on-site feed mill and contained (DM basis) steam-flaked corn (55.2%), Sweet Bran Plus (17.9%; Cargill Corn Milling), wet distiller’s grains plus solubles (17.2%), cotton burrs initially, then ground corn stalks (7.2%), corn oil (1.5%), glycerin (1.0%), and microingredients (0.03%). Monensin sodium (Rumensin, Elanco Animal Health) was included in the RAMP (20.0 g/907 kg) and finish diet (40.0 g/907 kg) throughout the experiment. Tylosin phosphate (Tylan, Elanco Animal Health) was included only in the finish diet at 9.2 g/907 kg. To control the outbreak of heifers cycling, MGA was included in the diet at 0.40 mg per heifer daily. All heifers were fed 27.3-g/907-kg ractopamine hydrochloride for approximately 31 d prior to slaughter (all feed additives on a DM basis). Samples of each diet were collected daily from the feed bunk and subsamples dried at 100 °C. Daily DM was averaged weekly and used for the calculation of DMI. The finishing diets provided protein and minerals to meet or exceed requirements (NRC, 1996).

Heifers were weighed as a group by pen using a platform scale on study day 0 and the morning of shipment for the calculation of live growth performance. Body weights were measured prior to the morning feeding and a 4% pencil shrink was applied to initial and final BW. Carcass-adjusted performance was calculated from HCW/DP in decimal form.

Trained personnel from the Beef Carcass Research Center recorded individual animal ear tag numbers in the sequence of harvest and affixed a harvest sequence number to each carcass. Plant carcass ID and HCW were recorded and verified by carcass sequence number. Carcasses were graded after approximately 36-h chill. United States Department of Agriculture QG (assigned by USDA Grader) and YG (assigned by camera system) and grading camera measurements were obtained from the packing plant records. The DP for each pen was calculated as the mean HCW/mean shrunk live weight (4% shrink) × 100.

### Statistical Analyses

Linear mixed models were used for all analyses using the GLIMMIX procedure of SAS 9.4 (SAS Inst., Inc., Cary, NC). Fixed effects included implant treatment, serial slaughter time, and interaction. No interaction was detected for any dependent variables (*P* > 0.10) except for DMI (*P* = 0.04; data not shown), as such, only the main effect of the implant is discussed in this analysis. Random intercepts were used to account for trial (location) and block within trials. Model-adjusted treatment means for each implant group and corresponding SEM are reported. Comparisons of the distributions of quality and YG data among treatment groups were analyzed in generalized linear mixed models for ordinal outcomes (cumulative logit, multinomial) with similar fixed effects as those described above (Osterstock et al., 2010). Random intercepts for pen (in addition to trial and block) were included since these data were analyzed on an individual outcome basis.

## RESULTS

Heifer growth performance, mortality, removals, and realizers are presented in Table 2. No significant differences were detected (*P* ≥ 0.11) for initial BW (333 vs. 333 ± 941 kg); final BW calculated with deads and removals—excluded (599 vs. 600 ± 4.5 kg), ADG (1.51 vs. 1.51 ± 0.022 kg) or gain-to-feed ratio (G:F; 0.160 vs. 0.159 ± 0.0010); final BW calculated with deads and removals—included analysis for ADG (1.44 vs. 1.44 ± 0.022 kg) or G:F (0.153 vs. 0.152 ± 0.0020); mortality (0.83 vs. 0.94 ± 0.160 %), removals (1.43 vs. 1.17 ± 0.220 %), or realizers (1.99 vs. 1.89 ± 0.270 %) for REPEATED and SINGLE, respectively. Daily DMI was influenced by implant program, and heifers administered the SINGLE implant had greater (*P* = 0.02) DMI (9.46 vs. 9.38 ± 0.127 kg) compared to heifers administered REPEATED.

**Table 2.** Model-adjusted mean finishing performance and health outcomes of heifers implanted with Revalor-IH followed by reimplant with Revalor-200 (REPEATED) or a single Revalor-XH at arrival (SINGLE)

	Implant program^*a*^			
Item	REPEATED	SINGLE	SEM	*P*-value
Initial BW, kg^*b*^	333	333	4.1	0.29
DMI, kg^*b*^	9.38	9.46	0.127	0.02
Deads and removals excluded				
Final BW, kg^*b*^	599	600	4.5	0.63
ADG, kg^*b*^	1.51	1.51	0.022	0.35
F:G^*b*^	6.27	6.30	0.045	0.30
G:F	0.160	0.159	0.0010	0.40
Deads and removals included				
ADG, kg^*b*^	1.44	1.44	0.022	0.92
F:G^*a*^	6.57	6.64	0.067	0.11
G:F	0.153	0.152	0.0020	0.24
Mortality^*b*^	0.83	0.94	0.160	0.59
Removals^*c*^	1.43	1.17	0.220	0.32
Realizers^*c*^	1.99	1.89	0.270	0.72

^*a*^Treatments were: Revalor-IH (80 mg TBA and 8 mg E_2_, uncoated) on arrival followed by Revalor-200 (200 mg TBA and 20 mg E_2_, uncoated) at approximately 70–101 DOF; or Revalor-XH (80 mg TBA and 8 mg E_2_, uncoated; 120 mg TBA and 12 mg E_2_, coated; 200 mg TBA and 20 mg E_2_, total) on arrival (SINGLE).

^*b*^Enrolled heifers = 17,675, pens = 180, blocks = 54, studies = 6; DOF range = 145–222, mean = 180 DOF.

^*c*^Enrolled heifers = 13,989, pens = 164, blocks = 46, studies = 5; DOF range = 150–222, mean = 183 DOF.

Carcass characteristics are presented in Table 3. Marbling scores did not differ (*P* = 0.74; 503 vs. 505 ± 5.2) for REPEATED and SINGLE heifers. Heifers in REPEATED had greater (*P* ≤ 0.02) DP (64.54 vs. 64.22 ± 0.120 %), HCW (384 vs. 382 ± 2.8 kg), and ribeye area (91.87 vs. 89.55 ± 0.839 cm^2^), as well as lesser (*P* ≤ 0.01) rib fat (1.78 vs. 1.83 ± 0.025 cm) and calculated YG (2.82 vs. 2.97 ± 0.040) compared to heifers administered SINGLE. The distribution of USDA YG was impacted (*P* = 0.03) by implant regimen: 6.61 % vs. 4.26 % for YG 1, 31.04 % vs. 25.71 % for YG 2, 43.24 % vs. 45.80 % for YG 3, 16.38 % vs. 20.75 % for YG 4, and 2.72 % vs. 3.48 % for YG 5 in heifers from REPEATED compared to SINGLE. Distribution of USDA QG also was altered (*P* = 0.01) by implant program: 5.41 % vs. 7.02 % for USDA Prime, 78.41 % vs. 79.70 % for USDA Choice, 14.47 % vs. 11.98 % for USDA Select, and 1.71 % vs. 1.30 % for all other possible grades in heifers administered REPEATED compared to SINGLE.

**Table 3.** Model-adjusted mean carcass characteristics of heifers implanted with Revalor-IH followed by reimplant with Revalor-200 (REPEATED) or a single Revalor-XH at arrival (SINGLE)

	Implant program^*a*^			
Item	REPEATED	SINGLE	SEM	*P*-value
Dressing, %^*b*^	64.54	64.22	0.120	0.01
HCW, kg^*b*^	384	382	2.8	0.02
Ribeye area, cm^2*c*^	91.87	89.55	0.839	0.01
Marbling^*c*,*d*^	503	505	5.2	0.74
Rib fat, cm^*c*^	1.78	1.83	0.025	0.01
Calculated YG^*e*^	2.82	2.97	0.040	0.01
USDA YG, %^*e*^				
1	6.61	4.26	—	0.03
2	31.04	25.71		
3	43.24	45.80		
4	16.38	20.75		
5	2.72	3.48		
USDA QG, %^*f*^				
Prime	5.41	7.02	—	0.01
Choice	78.41	79.70		
Select	14.47	11.98		
Other	1.71	1.30		

^*a*^Treatments were: Revalor-IH (80 mg TBA and 8 mg E_2_, uncoated) on arrival followed by Revalor-200 (200 mg TBA and 20 mg E_2_, uncoated) at approximately 70 to 101 DOF; or Revalor-XH (80 mg TBA and 8 mg E_2_, uncoated; 120 mg TBA and 12 mg E_2_, coated; 200 mg TBA and 20 mg E_2_, total) on arrival (SINGLE).

^*b*^Carcasses = 17,167, pens = 180, blocks = 54, studies = 6; DOF range = 145–222, mean = 180 DOF.

^*c*^Carcasses = 13,895, pens = 126, blocks = 45, studies = 5; DOF range = 145–192, mean = 171 DOF.

^*d*^Small^00^ = 400.

^*e*^Carcasses = 17,159, pens = 180, blocks = 54, studies = 6; DOF range=145–222, mean = 180 DOF.

^*f*^Carcasses = 17,160, pens = 180, blocks = 54, studies = 6; DOF range = 145–222, mean = 180 DOF.

## DISCUSSION

A primary challenge to using steroidal implants to improve cattle growth performance is the payout period of steroidal hormone from the implant pellets. Payout-period duration from cholesterol and polyethylene glycol excipient matrices is typically from 60 to 120 d following implantation (Mader, 1998; Smith et al., 2018). The DOF for most feedlot cattle is 201 d (Samuelson et al., 2016). Reimplantation of cattle in the feedlot allows for cattle to have hormonal payout throughout the entire feeding period when using implants that have a payout period on average of approximately 90 d (Mader, 1998; Smith et al., 2018). In recent years, the use of coated implants has become an alternative for producers who do not want to reimplant cattle during the time cattle are on feed. Coated implants come in a variety of doses, polymer coating types, and ratios of androgen to estrogen (Smith and Johnson, 2020). Reimplanting must be done properly to avoid undue stress on cattle (Stanton, 1997). A reduction in DMI can result in negative effects on cattle growth performance and carcass traits and increase the cost of BW gain (Stanton, 1997). If these stresses and labor concerns can be minimized, reimplantation has additional benefits for producers who perform terminal sorting or have the ability to reimplant cattle in a low-stress manner. These benefits include lower product cost of noncoated implants compared to coated implants and also gives producers the flexibility to market cattle within a given pen as they become market ready, thus reducing the impact of carcass discounts received for overly finished or heavy carcasses that are subject to discount from the packer.

Combination TBA + E_2_ implants have been demonstrated to increase the final BW by upward of 30 kg over nonimplanted cattle (Guiroy et al., 2002; Parr et al., 2011a, 2011b; Smith et al., 2018). Also, cattle receiving more than one combination of TBA + E_2_ implant during the feedlot phase of production or greater total doses of TBA + E_2_ throughout the feedlot production phase have shown improvements in animal growth performance and HCW (Reinhardt and Wagner, 2014). While a negative control treatment was not included in any of the current experiments used to conduct the pooled analysis in the present study, all experiments provide evidence that greater total doses of TBA and E_2_ did not improve any live animal growth performance parameters. This finding is inconsistent with what some have demonstrated previously (Wileman et al., 2009; Reinhardt and Wagner, 2014). However, the current results are similar to others (Guiroy et al., 2002; Hilscher et al., 2016; Ohnoutka et al., 2018) that also have documented that greater total doses of anabolic steroid hormones in steers (Hilscher et al., 2016) and heifers (Ohnoutka et al., 2018) do not alter final BW, ADG, or G:F. Additionally, it has been previously demonstrated that steers administered a coated implant (initial and delayed-release implant formulation), Revalor-XS (Merck Animal Health, 200 mg TBA and 40 mg E_2_) 213 d prior to harvest had greater final BW, ADG, and improved G:F compared to steers administered a single Revalor-200 (Merck Animal Health, 200 mg TBA and 20 mg E_2_) at either 213 or 143 d prior to harvest (Smith et al., 2018), indicating the benefits in performance when cattle are exposed to hormone payout over the entirety of the feeding period. Reasons for greater total doses of steroid hormones in heifers not resulting in improved performance responses could be attributed to greater levels of endogenous E_2_ in circulation compared to steers (Heitzman, 1976) or the fact that absolute growth response in heifers is lesser than steers due to a smaller response surface (Smith and Johnson, 2020). This reduced the effect of the added hormone in heifers also could be an effect of heifers having a lower muscle-to-fat ratio and a lower basal growth rate than steers and, therefore, an effect of any growth-promoting technology will be less in heifers than steers (Smith et al., 2020). The use of a nonimplanted group of cattle can assist in better understanding the observed growth responses between varying implant regimens, and these comparisons are more readily attainable in small-pen feedlot research facilities due to lesser economic losses having fewer nontreated animals. Dry matter intake was increased in SINGLE heifers over reimplanted heifers, and heifers from SINGLE received less TBA and E_2_ compared to REPEATED. When single and repeated implants (equal total dose) were administered to beef steers, no reduction in cumulative DMI was noted (Parr et al., 2011b). In some cases, there has been evidence of decreased intake following reimplanting; however, this manifestation of intake reduction has never been demonstrated in small-pen studies where cattle are more routinely weighed and accustomed to the weighing and processing procedure. This reduction in DMI could be due to a variety of other factors, including the number of cattle processed or implanted on a given day, time away from the home pen, or other factors in commercial production settings that cannot be simulated in small-pen research facilities. The reduction in DMI finding deserves further investigation in order to fully understand the mechanism of action in which reimplanting cattle moderately reduces cattle intake. The increase in DMI (0.08 kg/d) by SINGLE heifers in the current analysis did not result in significant differences in ADG or G:F.

Reimplanted heifers had greater HCW compared to single-implant heifers in the present analysis. It has been reported that greater total doses of anabolic steroids do not increase HCW in steers or heifers (Hilscher et al., 2016), while others have demonstrated that this is not the case in steers (Smith et al., 2018). Heifers administered the REPEATED treatment had increased DP in the present analysis. Reinhardt and Wagner (2014) reported that greater doses of steroid hormones improve DP in steers and heifers; alternatively, others have demonstrated that greater total doses of anabolic steroids do not alter DP in cattle (Hilscher et al., 2016). Differences in DP were expected in this pooled analysis with an improvement in HCW but no impact to final BW. Previous research by Hilscher et al. (2016) may have failed to observe a difference in HCW and DP due to influences of greater total doses of steroid hormones on membrane-bound steroid hormone receptors and receptor desensitization (Smith et al., 2019b). This is in contrast to the present analysis in that greater total doses of anabolic compounds increased HCW and DP in heifers. There are distinct advantages to a pooled analysis versus a single-trial analysis, such as a greater sample size to compare outcomes and also that a pooled analysis provides estimated means and the associated SEM with more potential generalizability as they represent data from multiple study populations, sites, breed types, and basal dietary ingredient composition. Implant regimen employed altered the proportion of carcasses grading USDA Prime, Choice, Select, or sub-Select in the present study. This finding is consistent with what has been demonstrated in steers given increasing doses of anabolic hormones (Hilscher et al., 2016). The use of greater doses of anabolic hormones in the present study resulted in alterations in the proportion USDA YG classifications. Alterations in USDA YG have been demonstrated previously in heifers administered increasing doses of anabolic steroids, but this response was not noted in steers (Hilscher et al., 2016).

## CONCLUSION

This pooled analysis indicates that heifer growth performance (ADG and G:F) and heifer outcome (mortality, removal, or realizers) are not altered by a single, coated implant or noncoated reimplant regimen given the DOF employed in the present analysis. Carcass measures of leanness and muscling were improved in REPEATED. These data may indicate that REPEATED may be suited for grid-based marketing, and SINGLE might be suited for heifers sold on a live basis depending upon market conditions and value-based grid premiums and discounts. However, these decisions are operational dependent and also may be influenced by factors including animal and employee safety, stress on animals, processing facilities, time of year, labor availability, and marketing strategies.
